# Personality subtypes in adults with social anxiety disorder - novelty seeking makes the difference

**DOI:** 10.1186/s12888-022-04484-z

**Published:** 2022-12-27

**Authors:** Man-Long Chung, Laura-Effi Seib-Pfeifer, Christina Elling, Franziska Geiser, Andreas J. Forstner, Johannes Schumacher, Rupert Conrad

**Affiliations:** 1 Department of Psychosomatic Medicine and Psychotherapy, University Hospital Bonn, Venusberg Campus 1, 53127 Bonn, Germany; 2 Department of Psychology, University of Bonn, Kaiser-Karl-Ring 9, 53111 Bonn, Germany; 3 Institute of Human Genetics, University of Bonn, School of Medicine & University Hospital Bonn, Venusberg Campus 1, 53127 Bonn, Germany; 4 Institute of Neuroscience and Medicine (INM-1), Research Centre Jülich, Wilhelm-Johnen-Straße, 52428 Jülich, Germany; 5 Centre for Human Genetics, University of Marburg, Baldingerstraße, 35033 Marburg, Germany; 6 Department of Psychosomatic Medicine and Psychotherapy, University Hospital Münster, Albert-Schweitzer-Campus 1, 48149 Münster, Germany

**Keywords:** Social Anxiety Disorder, Temperament, Personality, Novelty Seeking, Harm Avoidance, Cluster Analysis

## Abstract

**Background:**

Up to now several subtypes of social anxiety disorder (SAD) have been proposed.

**Methods:**

In the present study, we used a cluster analytic approach to identify qualitatively different subgroups of SAD based on temperament characteristics, that is, harm avoidance (HA) and novelty seeking (NS) dimensions of Cloninger’s Temperament and Character Inventory.

**Results:**

Based on a large, diverse clinical sample (*n* = 575), we found evidence for two distinct subgroups of SAD: a larger (59%) prototypic, inhibited cluster characterized by high HA and low NS, and a smaller atypic, and comparatively more impulsive cluster characterized by medium to high HA and increased NS. The subgroups differed regarding a variety of sociodemographic and clinical variables. While the prototypic SAD subtype suffered from more severe SAD and depressive symptoms, suicidal ideation, and reduced social functioning, the atypic NS subtype showcased higher reproductive behaviour, self-directedness and -transcendence, comparatively. Additional hierarchical logistic regression highlights the contribution of age and education.

**Conclusions:**

Our results valuably extend previous evidence for the existence of at least two distinct subtypes of SAD. A better knowledge of the characteristic differences in prototypic behaviour, personality, coping strategies and comorbidities between the identified (and further) subtypes can contribute to the development of effective prevention interventions and promotes the conceptualization of tailored treatments.

**Supplementary Information:**

The online version contains supplementary material available at 10.1186/s12888-022-04484-z.

## Background

Social anxiety disorder (SAD) is among the most common anxiety disorders [1]. It is characterized by a marked fear and avoidance of social situations that involve the potential for evaluation or rejection by others. SAD is associated with high rates of comorbidity, in particular with avoidant personality disorder (APD), affective and other anxiety disorders [2, 3]. It is further related to high psychosocial impairment and discussed as a risk factor for depressive and substance use disorders [1, 2, 4].

Despite the disorder-defining characteristics that all individuals with SAD share, several subtypes have been proposed and debated [5, 6]. These subtypes show different phenotypes and are proposed to represent different etiological subgroups, including the genetic factors involved [7]. For instance, based on symptomatology one can differentiate a generalized subtype characterized by fear/avoidance of a broad array of social situations from a subtype with the predominant fear of displaying physical signs of anxiety (see DSM-IV; [8]). This distinction, however, is under critical debate, and is not part of the social phobia characterization raised in DSM-5. In individuals with a performance-only anxiety subtype as classified in DSM-5, anxiety is limited to speaking or performing in public situations [9]. SAD subtypes are further differentiated based on comorbid psychiatric disorders such as alcohol use disorder [10, 11] or, with respect to axis-II, APD [12].

Besides above-mentioned distinctions, more recently, a categorization of SAD subtypes based on the Big-5 personality traits was proposed, consisting of three clusters (prototypical, introvert-conscientious, instable-open) [13]. Especially neuroticism and extraversion are relevant for this categorization. Interestingly, two temperament dimensions, harm avoidance (HA) and novelty seeking (NS) [14, 15], which have been connected to the aforementioned personality traits [16, 17], are of particular interest as well [18, 19]. According to the psychobiological model of personality of Cloninger et al. [15], one can distinguish between three character dimensions (cooperativeness, self-directedness, and self-transcendence), and four temperaments (HA, NS, persistence and reward dependence) that are all supposed to have their corresponding biological substrate. The dimensions can be measured via the well-established “Temperament and Character Inventory” (TCI) questionnaire [15, 20].

Heritability estimates for the TCI scales range from 30–60% [21, 22]. During the past decades, the theory driven conceptualisation of the TCI-temperament and -character dimensions has been discussed and evaluated by a variety of phenotypic and genetic studies [21–27]. The proposed temperament traits have been found to be associated with several psychiatric disorders. For instance, high levels of HA and avoidance tendencies have been linked to major depression disorder (MDD) [28, 29] and several anxiety disorders [18, 19, 29, 30] while NS and behavioural approach in Eysenck’s model were associated with substance use disorders [25, 31], and, inversely, also anxiety disorders [19, 29, 30]

Focussing on HA and NS, both mirror differences in the automatic emotional response to stimuli. HA, most likely regulated by the serotonin system, is associated with inhibited behaviour and the tendency to respond fearfully to novel situations. In contrast, NS, regulated by the dopamine system, describes a tendency to respond actively to novel stimuli and is associated with impulsive and exploratory behaviour [14, 15]. In line with the prototypical SAD phenotype characterized by behavioural inhibition, avoidance of social situations and shyness are associated with increased HA [18, 32–39], and reduced NS [32, 35, 36]; for meta-analytic findings see [19].

However, there is also evidence for a more atypical subgroup of SAD, still affected by social anxiety, but simultaneously characterized by impulsive, exploratory and risky behaviour, reflecting increased NS [7, 36, 40–43]. Individuals in the high NS subtype are more often affected by comorbid impulse control, bipolar [41] and substance use disorders [7, 44], suffer more often from severe substance use [42] and greater functional impairment, are less educated and of younger age [41]. Furthermore, there is some evidence that they are less likely to seek for, complete, or fare well in treatment [43]. Taking a closer look at lifetime impulsive control problems, Binelli et al. [7] reported (descriptive) differences between the two subtypes with more frequent suicide attempts and self-harm in the impulsive high NS subgroup. They further differentiated the pattern of substance use and reported a greater use of alcohol in the inhibited/low NS subgroup, as compared to a more pronounced use of substances with a higher-sensation-seeking profile in the impulsive/high NS subgroup. However, this more differentiated pattern of substance use seems to be specific for the analysed sample and was not reported in other studies [44]. Furthermore, reverse findings with increased alcohol use and stronger craving in more impulsive SAD patients have also been reported [45]. Cluster research focusing on the Big-5 personality traits also identified an anxious-extravert subtype characterized by high neuroticism, and simultaneously high levels of extraversion, impulsivity, excitement seeking, and monotony avoidance [13].

In sum, the differentiation of these two SAD subtypes – both characterized by high HA but one displaying low and the other high NS – was repeatedly mirrored in cluster analytic approaches [7, 40, 42, 46] and latent class analyses [41, 47]. With respect to the proposed subtype-characteristics, it needs to be noted that the differences reported in previous studies are still inconsistent. They are partly based on rather homogenous, small (e.g. [7]: *n* = 142; [42]: *n* = 82; [40]: *n* = 125; [46]: *n* = 84), often mere student’s samples [7, 40]. Beyond that, most previous work neglected potential differences in relevant comorbidities such as anxiety disorders other than SAD, and MDD, or former psychotherapeutic treatment. Therefore, further replication and exploration in large, heterogeneous, clinical samples is needed [18].

A profound characterization of SAD subtypes based on distinct personality profiles can contribute to a valid identification and diagnosis of SAD patients and promotes the conceptualization of tailored treatments [43]. With this in mind, the present study utilised a cluster analytic approach to identify and validate qualitatively different subgroups of SAD based on HA and NS in a large (*n* = 575) sample of SAD patients. Cluster analysis revolved around the TCI temperament scales HA and NS due to meta-analytic evidence [19] and existing research [7, 40, 42, 46]. We expect to find the best fit for a two-cluster solution differentiating two distinct subtypes: a larger subgroup that shows the prototypic inhibiting SAD pattern with high HA and low NS and a smaller, atypic subgroup with impulsive tendencies, that is, high HA and high NS. Previous research suggests that the two subtypes may differ regarding several sociodemographic and clinical variables. However, based on the rather scarce data basis, we refrain from any specific predictions. Additionally, due to potential implications for therapeutic interventions, personal skill training for SAD individuals and the lack of multivariate research, we conduct a hierarchical logistic regression to identify sociodemographic and psychiatric predictors for cluster affiliation.

## Methods

### Sample

The final sample consisted of *n* = 575 [*n*_*female*_ = 337 (59%); age: 18–78 years; *M* = 41.60, *SD* = 13.96] German participants with a DSM-IV lifetime diagnosis of SAD as confirmed by the Structured Clinical Interview for DSM-IV axis-I disorders (SCID-I) [48, 49]. All participants showed clinical significant social anxiety as indicated by a score of 19 or above in the Social Phobia Inventory (SPIN) [50, 51]. Recruitment was realised within the context of the German “Social Phobia Research” project which was set up in 2012 as a collaboration between the Department of Psychosomatic Medicine and Psychotherapy and the Institute of Human Genetics at the University of Bonn, Germany. Participants were recruited via various access paths (e.g., clinical services, cooperation with external clinics and outpatient practitioners, internet, reports on regional TV and radio channels, newspaper advertisements and articles, advertisements on urban areas). Exclusion criteria were a comorbid psychosis, inadequate German language skills or somatic and/or mental difficulties in completing inserted questionnaires. The study was approved by the local ethics committee of the University of Bonn and was in accordance with the declaration of Helsinki. All participants gave written informed consent.

### Measures

Demographic information was assessed by a standardised questionnaire. This included sex, age, current partnership status and level of education. Furthermore, family psychiatric history and previous treatment for mental disorders were enquired.

Diagnoses of SAD and relevant psychiatric comorbidities, including MDD, anxiety disorders, posttraumatic stress disorder (PTSD), and substance use disorders (alcohol, cannabis, other substances), were assigned by the German version of the SCID-I while APD was assessed by the German version of the SCID-II [48, 49] administered by trained interviewers. Due to economic reasons and the high comorbidity between SAD and APD, only the APD section of the SCID-II was considered. Interrater-reliability for SCID-I ranges from kappa values 0.61 and 0.83 and for SCID-II, a kappa value of 0.83 for APD was achieved [52].

Severity of social anxiety symptoms was measured by the German version of the SPIN [50, 51]. As a valid brief self-report measure, the SPIN captures behavioural, physiological, and cognitive symptoms of social anxiety on 17 items using a four-point Likert-scale ranging from *not at all* to *extremely*. The total sum score ranges between 0 and 68. Total scores of 19 and above indicate clinical relevant social anxiety [50].

The German version of the Beck Depression Inventory (BDI) [53, 54] served as a measure of severity of potential comorbid depressive symptoms. On a four-point Likert scale (0–3), 21 items ask participants for a self-report of past weeks depressive symptoms. Sum scores range from 0 to 63. The BDI provides a highly valid and reliable measure of depressive symptoms [20]. As a short measure of suicidality, we further analysed item I of the BDI that explicitly asks participants for any suicidal ideations.

Personality was assessed by the German version of the TCI [15, 55], a valid, psychological and neurobiological based, measure of temperament and character. Using 240 items, the TCI captures four temperaments (HA, NS, persistence, and reward dependence) and three character dimensions (cooperativeness, self-directedness, and self-transcendence) by means of a dichotomous true/false format. T-scores for each scale was utilised, which are based on norm values from healthy subjects.

To verify the validity of the TCI-based SAD-clusters, we further included a short measure of behavioural inhibition and behavioural approach [56–59]. On a theoretical level, HA and the behavioural inhibition system (BIS) and NS and the behavioural approach system (BAS) share several properties [60, 61]. Accordingly, the atypic-impulsive subtype should be characterized by increased BAS, while the prototypic-inhibited subtype should exhibit increased BIS. The applied short version of the action regulating emotions system scales (ARES scales) measures BIS and BAS sensitivity by means of 20 four-point Likert-scaled items [62]. Reliability (Cronbach’s α) of all questionnaires was good to excellent, with few exceptions among subscales (see Table 1).

**Table 1:** Reliability of the applied questionnaires in the given data

	SPIN	BDI	TCI							ARES	
			NS	HA	RD	P	SDi	C	ST	BIS	BAS
Cronbach’s α	.88	.90	.85	.82	.71	.47	.87	.85	.85	.90	.59

*SPIN* Social phobia inventory, *BDI* Beck depression inventory, *TCI* Temperament and character inventory, *NS* Novelty seeking, *HA* Harm avoidance, *RD* Reward dependence, *P* Persistence, *SDi* Self-directedness, *C* Cooperativeness, *ST* Self-transcendence, *ARES* Action regulating emotions system, *BAS* Behavioural approach system, *BIS* Behavioural inhibition system

### Statistical analyses

To identify personality-based subgroups of SAD, the global T-Scores of HA and NS were submitted to a hierarchical cluster analysis using Ward’s method and squared Euclidean distance. The number of clusters was determined by a dendrogram which served as the basis for a subsequent k-means cluster analysis. The formal evaluation of the adequate number of clusters was based on the formal fit indicators ETA^2^, PRE, and FMX [63]. Cluster solutions containing two to six clusters were analysed. The best fitting cluster solution was evaluated by stability checks using Cohen’s kappa [64]. Internal validity of the clusters was evaluated with respect to BIS and BAS scales.

Potential differences between the identified clusters regarding sociodemographic-, psychiatric-, and personality variables were examined using *t-*tests for quantitative variables and chi^2^-tests or Fisher’s exact test for categorical data. Multiple testing was accounted for by adjusting the *p*-value utilising the Benjamini–Hochberg procedure with a false discovery rate of 0.05 [65]. Based on 63 significance tests, critical values were calculated for each *p*-value. After identifying the largest *p*-value that is still under the critical value (here: *p* = 0.011, Benjamini–Hochberg critical value = 0.021), all results with lower *p-*values were considered significant. Cohen’s *d* and Cramer’s *V* served as measures of effect size.

A hierarchical logistic regression with cluster type as the dependent variable was further conducted as suggested by one of the reviewers. Sociodemographic variables (age, sex, partnership, education, smoking status, medication) were entered first followed by psychiatric comorbidities (panic disorder, agoraphobia, generalized anxiety disorder, specific phobia, posttraumatic stress disorder, major depression disorder, APD, alcohol- and substance-related disorders). A correlation matrix for all predictors detected no multicollinearity. Goodness-of-fit was evaluated by the Hosmer–Lemeshow-Test while Nagelkerke’s R^2^ represented the explained variance by the predictors. All analyses were performed using SPSS Version 27 [66].

## Results

### Results of the hierarchical cluster analysis

The combined analysis of formal fit indicators revealed the best fit for a two-cluster solution which explained 46% of variance (ETA^2^ = 0.46) and showed best PRE (PRE = 0.46) and FMX (FMX = 404.09) values (for a direct comparison with alternative three- to six-cluster solutions, see supplementary table A). The two-cluster solution was further supported by visual inspection of plotted fit-values with clearest peaks at the two-cluster solution. Stability checks based on Cohen’s kappa revealed an assignment of cases to clusters that was independent of the starting values. That is, the two-cluster solution proofed high stability with all Cohen’s kappa >|.940|.

The first, larger cluster was characterized by very high HA (+ 2 standard deviation (SD)) and low NS (- 1 SD) T-scores. 59% (*n* = 338) of the sample were assigned to this prototypic cluster. The second, smaller cluster was characterized by high HA (+ 1 SD) and average NS T-scores. 41% (*n* = 237) of the sample were assigned to this atypic cluster (see Table 2).

**Table 2:** Comparison of TCI dimensions, sub dimension and BIS/BAS scores between SAD subtype groups

	Prototypic ↑↑HA / ↓NS	Atypic ↑HA / NS			
	*n* = 338 (59%)	*n* = 237 (41%)			
	mean (*SD*)	mean (*SD*)	*t*	*p*^a^	*d*
Harm Avoidance	74.48 (6.79)	62.19 (9.50)	18.09	**< .001**	1.53
anticipatory worry	69.84 (9.27)	56.30 (10.89)	15.59	**< .001**	1.36
fear of uncertainty	61.49 (5.72)	54.31 (10.13)	9.86	**< .001**	0.92
shyness	67.53 (5.32)	61.88 (8.61)	8.97	**< .001**	0.82
fatigability	72.65 (10.45)	63.14 (12.62)	9.53	**< .001**	0.84
Novelty Seeking	35.50 (7.76)	53.51 (9.49)	24.97	**< .001**	2.12
exploratory excitability	32.11 (9.65)	46.02 (10.41)	16.48	**< .001**	1.40
impulsiveness	40.92 (7.90)	53.45 (12.96)	13.26	**< .001**	1.22
extravagance	43.19 (11.42)	55.52 (13.06)	12.00	**< .001**	1.02
disorderliness	46.57 (9.45)	54.39 (10.47)	9.33	**< .001**	0.79
Reward Dependence	49.34 (10.67)	50.30 (10.74)	1.06	.145	0.09
Persistence	52.29 (10.72)	49.79 (12.71)	2.47	**.007**	0.21
Self-Directedness	28.71 (13.69)	34.97 (14.81)	5.22	**< .001**	0.44
Cooperativeness	43.95 (14.08)	45.01 (14.57)	.87	.190	0.07
Self-Transcendence	45.06 (10.75)	48.71 (12.48)	3.66	**< .001**	0.32
BIS	11.98 (2.43)	10.35 (2.87)	7.12	**< .001**	0.62
BAS	8.49 (1.93)	9.33 (1.94)	7.07	**< .001**	0.44

*HA* Harm avoidance, *NS* Novelty seeking, *n* sample size, *SD* standard deviation, *t* t-Test, *d* Cohen’s d, *BIS* Behavioural inhibition system, *BAS* Behavioural activation system

^a^significant *p*-values after Benjamini–Hochberg adjustment are in bold

The internal validity of the two-cluster solution was further proved by the pattern of differences in BIS and BAS sensitivity. In line with our predictions, the prototypic cluster showed high BIS and lower BAS values, while the atypic cluster was characterized by still high, but compared to the prototypic cluster, reduced BIS and higher BAS values.

### Subtype comparison

Total NS and all NS subscale T-scores were greater in the atypic compared to the prototypic cluster. For HA, the pattern of results was reverse, with higher total HA and HA subscale T-scores in the prototypic compared to the atypic cluster (Table 2). Concerning the other TCI subscales, higher T-scores in “self-directedness” and “self-transcendence” were reported in the atypic cluster while the prototypic cluster achieved higher T-scores in “persistence”.

The prototypic cluster contains younger and more SAD individuals with children than the prototypic cluster but there were no significant differences regarding other sociodemographic variables (see Table 3). However, individuals in the prototypic cluster descriptively showed reduced levels of social functioning as indicated by less partnerships and reduced education. They further reported significantly higher symptom severity as measured by the SPIN. While there were no differences regarding former psychotherapeutic treatment of SAD, an increased number of individuals in the prototypic cluster reported a history of former medical treatment.

**Table 3:** Comparison of empirical clusters based on TCI-NS and TCI-HA dimensions

	Prototypic ↑↑HA / ↓NS	Atypic ↑HA / NS	*t / χ*^*2*^* / Fisher’s exact test*^*1*^	*p*^a^	*d /* ϕ
	*n* = 338 (59%)	*n* = 237 (41%)			
	*n* (%) / mean (*SD*)	*n* (%) / mean (*SD*)			
Sociodemographics					
sex (female)	202 (59.8%)	135 (57%)	0.41	.521	.03
age	38.56 (12.58)	45.95 (14.70)	6.30	**< .001**	.55
current partnership (yes)	151 (46.7%)	124 (56.1%)	4.60	.032*	.09
education (at least high school)	208 (61.5%)	157 (66.2%)	1.33	.249	.05
children (yes)	86 (25.7%)	114 (48.5%)	31.63	**< .001**	.24
smoking (yes)	67 (19.8%)	52 (21.9%)	0.55	.456	.03
SAD characteristics					
SPIN	43.85 (10.33)	38.01 (10.40)	6.65	**< .001**	.56
SAD generalized	275 (86.2%)	190 (86.4%)	0.69	.707	.04
Former SAD treatment					
Psychotherapeutic / psychiatric	172 (50.9%)	108 (45.6%)	1.58	.209	.05
medical	96 (68.6%)	42 (47.7%)	9.82	**.002**	.21
Family psychiatric history					
Any psychiatric disorder	105 (31.1%)	88 (37.1%)	2.30	.129	.06
SAD	15 (4.4%)	16 (6.8%)	1.46	.227	.05
Comorbidities ^b^					
Panic disorder	94 (28.1%)	61 (26.4%)	0.19	.665	.02
Agoraphobia	125 (37.5%)	82 (35.2%)	0.33	.569	.02
GAD	57 (17.0%)	29 (12.3%)	2.37	.123	.06
Specific phobia	101 (30.1%)	77 (32.8%)	0.47	.492	.03
Any anxiety disorder	209 (61.8%)	141 (59.5%)	0.86	.320	.02
PTSD	5 (1.5%)	2 (0.8%)	N/A^1^	.706	.03
MDD	272 (80.5%)	172 (72.9%)	4.57	.032*	.09
APD	251 (74.5%)	137 (58.1%)	25.66	**< .001**	.21
Alcohol-related disorder	74 (21.9%)	65 (27.4%)	2.33	.127	.06
Substance-related	78 (23.1%)	68 (28.7%)	2.32	.128	.06
Substance-related (cannabis)	1 (0.17%)	5 (0.87%)	N/A^1^	.087	.09
BDI	22.77 (11.39)	17.02 (9.87)	6.29	**< .001**	.53
BDI Item I	0.68 (0.68)	0.46 (0.66)	3.94	**< .001**	.33

*n* sample size, *SD* Standard deviation, *t* t-Test, *χ*^*2*^ chi-square, *p p*-Value, *d* Cohen’s d, ϕ Cramer’s V, *SAD* Social anxiety disorder, *SPIN* Social phobia inventory, *GAD* Generalized anxiety disorder, *PTSD* Posttraumatic stress disorder, *MDD* Major depression disorder, *APD* Avoidant personality disorder, *BDI* Beck depression inventory, *BDI* Item I = 0”I do not have any thoughts of harming myself; 3”I would kill myself if I could”, *TCI* Temperament and character inventory, *HA* Harm avoidance, *NS* Novelty seeking, *N/A* not available

*n* (%) refers to % within respective cluster

^*^ nominally significant *p* < .05

^a^ significant *p*-values after Benjamini–Hochberg adjustment are in bold

^b^ results refer to observed lifetime comorbidities

There were no significant differences regarding family psychiatric history (neither in general, nor specifically for SAD). Individuals in both clusters showed a similar amount of current and lifetime comorbid anxiety disorders (panic disorder, agoraphobia, generalized anxiety disorder, specific phobia) and PTSD. However, individuals in the prototypic cluster were more often affected by a comorbid lifetime MDD (nominally significant), reported higher BDI scores, and had higher score on the suicidality-item I of the BDI. Furthermore, lifetime APD was overrepresented in the prototypic cluster. With respect to current and lifetime substance use behaviour, there was a higher number of individuals in the atypic cluster that was affected by comorbid lifetime alcohol abuse and lifetime cannabis abuse, but no significant difference could be observed.

### Hierarchical logistic regression

The Hosmer–Lemeshow-Test yielded no significance for this logistic model, representing a good model fit (χ^2^(8) = 1.57, *p* = 0.99). Correct classification of SAD individuals based on included predictors lies at 65.6%. Both models overall achieved significance compared to a model without any predictors (Step 1: χ^2^(6) = 44.81, *p* =  < 0.001, Step 2: χ^2^(15) = 53.535, *p* =  < 0.001), however, if we look at the sole contribution of Step 2, the included predictors did not significantly improve the model (χ^2^(9) = 8.73, *p* = 0.46). A similar result can be found for each predictor in Step 2, as none of them can significantly predict the odds of being in the prototypic or atypic cluster (Table 4). In both models, age and a German high school degree reached significance after Benjamini–Hochberg adjustment. With each year, the odds of being in the atypic cluster is increased by 4%. Likewise, having a German high school degree increases the odds for the atypic cluster by over 40%. Altogether, the models explain 12% and 14% of the variance (Nagelkerke’s R^2^).

**Table 4:** Hierarchical logistic regression (*n* = 503)

Covariates	*B*	*SE B*	Wald	*p*^*a*^	*OR*	95% *CI*		*R*^2^
						***LL***	***UL***	
**Step 1**								
Age	-.043	.008	31.630	**< .001**	0.96	0.94	0.97	
Sex (female)	.038	.195	.038	.846	1.04	0.71	1.52	
Education (at least high school)	-.584	.211	7.636	**.006**	0.56	0.37	0.84	
Partnership (yes)	-.396	.192	4.238	.040*	0.67	0.46	0.98	
Medication (yes)	.085	.210	.163	.687	1.09	0.72	1.64	
Smoking (yes)	-.432	.234	3.420	.064	0.65	0.41	1.03	
								.12
**Step 2**								
Age	-.043	.008	28.450	**< .001**	0.96	0.94	0.97	
Sex (female)	-.051	.205	.062	.804	0.95	0.64	1.42	
Education (at least high school)	-.551	.216	6.491	**.011**	0.58	0.38	0.88	
Partnership (yes)	-.385	.197	3.818	.051	0.68	0.46	1.00	
Medication (yes)	.014	.220	.004	.949	1.01	0.66	1.56	
Smoking (yes)	-.380	.244	2.427	.119	0.68	0.42	1.10	
Panic disorder	.012	.275	.002	.965	1.01	0.59	1.73	
Agoraphobia	.203	.256	.627	.429	1.23	0.74	2.03	
GAD	.318	.289	1.205	.272	1.37	0.78	2.42	
MDD	.261	.241	1.167	.280	1.30	0.81	2.08	
Specific phobia	-.132	.216	.375	.541	0.88	0.57	1.34	
PTSD	.594	.900	.436	.509	1.81	0.31	10.58	
APD	.745	.674	1.221	.269	2.11	0.56	7.90	
Alcohol-related disorder	-.101	.237	.182	.669	0.90	0.57	1.44	
substance-related disorder	-1.014	.583	3.027	.082	0.36	0.12	1.14	
								.14

Target variable: cluster; Atypic cluster = 0, Prototypic cluster = 1

*n* sample size, *B* regression coefficient, *SE b* standard error regression coefficient, *p* p-value, *OR* odds ratio, *d* Cohen’s *d*, *CI* Confidence interval, *LL* Lower limit, *UL* Upper limit, *R*^*2*^ Nagelkerke’s R^2^, *GAD* Generalized anxiety disorder, *PTSD* Posttraumatic stress disorder, *MDD* Major depression disorder, *APD* Avoidant personality disorder

^*^ nominally significant *p* < .05

^a.^significant *p*-values after Benjamini–Hochberg adjustment are in bold

## Discussion

In the present study, we used a cluster analytic approach to characterize subtypes of SAD patients based on temperament traits of the TCI (HA and NS) in a large, diverse clinical sample (*n* = 575). In line with previous findings, we identified two distinct clusters, a larger subgroup of SAD patients showed a quite prototypic pattern with very high HA and low NS. In contrast, a smaller group of patients was characterized by high HA and average NS. These findings support previous reports of an atypic, more NS orientated subtype of SAD [7, 40–42, 46]. The validity of the cluster solution was further confirmed by the pattern of differences in BIS and BAS sensitivity. The two subtypes markedly differed with respect to several sociodemographic and clinical variables. These qualitative differences may entail critical implications for treatment approaches and treatment success [36, 43, 46].

Overall, female participants were overrepresented in our sample (59% female). However, this unbalanced sex distribution is well in line with epidemiological findings on the sex ratio of psychiatric diseases and SAD [67, 68]. There were no sex differences between the two clusters. Participants in the prototypic cluster were younger and, in tendency, showed increased daily life impairments as indicated by reduced education and a reduced probability of being in a partnership. They showed higher SAD symptom severity and more likely reported a history of former psychopharmaceutic treatment. Prototypic SAD patients more often suffered from a comorbid lifetime MDD, reported more severe depressive symptoms and suicidal ideation, and were more often affected by a comorbid APD. In contrast, participants in the atypic cluster showcased higher reproductive behaviour, self-directedness and -transcendence.

Thus, our data suggest the existence of two distinct subtypes of SAD: a prototypic subtype with more severe SAD and depressive symptoms that more often suffers from comorbid MDD, APD and increased social impairment. This *depressive subtype* is younger, and, in line with the increased symptom severity, more likely receives psychopharmacological treatment and reports more suicidal ideation. Conversely, the *NS subtype* shows less severe SAD and depressive symptoms, and a higher level of social functioning and reproductive behaviour.

In our sample, we found reduced symptom severity in the atypic cluster. This is well in line with previous reports of SAD symptom severity being positively related to HA [18, 19, 33, 37], and related concepts such as neuroticism [13], while negatively associated with NS [19], and related concepts such as openness, extraversion or impulsivity [13]. While increased HA implies a rather cautious, restrained behaviour, increased NS entails an approach tendency towards and increased interest in new, unknown stimuli [14]. This might also include anxiety-associated social situations. In terms of habituation, this NS-initiated self-exposure can serve as a functional self-regulatory approach reducing anxiety/SAD symptoms. Even though initially conceptualized as a stable trait, one might further speculate that this tendency towards an average NS behavioural strategy develops over the course of the SAD impairment, and, therefore, is more present in SAD participants of older age. This speculation would fit with the older age observed in the NS subtype.

Diverging from our results, previous studies did not report any differences in symptom severity [7, 42]. This might be due to rather small sample sizes and reduced variance in previous studies that only included specific SAD-subtypes [42] or patients that reported high symptom severity [7]. In contrast, striving to mirror the entire range of clinically relevant SAD symptoms, we included all participants with a clinically relevant SAD diagnosis in our study. At the same time, however, external validity might not be given for SAD individuals who showcase rather low symptom severity or those, who are only afraid of certain social situations as the distribution of generalized SAD was the same for both clusters.

Together with SAD symptom severity, participants in the atypic cluster less often suffered from comorbid MDD and showed reduced depressive symptom severity as measured by the BDI. These results fit with previous findings that showed reduced HA, as it was observed in the atypic cluster, to be associated with reduced depressive symptoms [37]. In line with that, a reduction of HA in the course of SAD treatment comes along with a significant reduction of depressive symptoms [34]. Even though HA is conceptualized as a stable trait, there is evidence that it might be affected by successful treatment of SAD [34, 36]. The same might be the case for NS. Based on the present findings, a modulation of HA and/or NS might provide a promising approach to be integrated in innovative SAD therapy. Crucially, higher HA has been linked to poorer outcome in different types of psychotherapies [18, 46, 69]. Reducing HA related safety behaviour that, combined with a comorbid APD, likely contributes to the maintenance of the disease [70], might reduce SAD severity. It could further increase responsiveness to cognitive behavioural therapy [36, 46].

Potentially, strengthening individual NS tendencies up to a certain degree could ease social interaction in everyday life, reduce functional impairment and increase the responsiveness for approach related therapeutic concepts. Reduced HA and increased NS behaviour in the atypic subtype may contribute to reduced symptom severity and thereby reduce the probability of burdening comorbidity with MDD and associated suicidal ideation. Then again, the overall reduced level of suffering allows higher levels of social functioning in the NS subtype. In contrast in the prototypic cluster, increased HA, conceptualized as an innate responsiveness to avoid punishment, may foster the development of SAD and secondary depressive symptoms. However, even though the proposed relations seem plausible, they remain rather speculative, as the cross-sectional approach of the present study prohibits any causal interpretation.

Despite potential protective effects of increased NS, this temperament dimension is also associated with impulsive, unpredictable behaviour [14]. Thus, it might increase harmful impulse-related behaviour such as self-harm, suicide attempts, and substance misuse [42]. In line with that, previous studies reported (at least a tendency towards) increased suicidal attempts and substance use in the identified atypic, high NS cluster [7, 42, 44]. However, there are also divergent findings. For instance, Kashdan and Hofmann [42] reported no differences in the occurrence of substance use disorder and the results in this present study also did not showcase increased substance or alcohol use in the atypic cluster. Based on our cross-sectional results, increasing NS tendencies to an average degree, comparable to healthy subjects, do not seemingly increase harmful impulse-related behaviour. Furthermore, considering the same distribution of generalized SAD in both clusters, presumably, NS tendencies do not influence the quantity but rather the handling of social situations in which SAD individuals face insecurities and anxiety.

Concerning substance use, it might function as a rather dysfunctional try of coping by creating safety, sedation or distraction when facing SAD symptoms [7, 42]. Such risky behaviour seems more likely in more impulsive, NS oriented individuals. In terms of alcohol it might be the case, that increased impulsivity promotes abuse and dependence, as it draws attention to the immediate, anxiolytic effects of alcohol instead of focusing potential negative (social) outcomes such as embarrassing behaviour [45]. Furthermore, reduced HA in the atypic cluster may come along with less pronounced protective behavioural strategies, that is, cognitive and behavioural harm-reducing strategies to decrease alcohol use and alcohol problems [4]. As our results do not support this evidence, in future studies, a longitudinal approach in combination with a dimensional, instead of the applied categorical measure of (subclinical) substance-related behaviour, would provide further inside into the proposed relations.

While substance use can be interpreted as a manifestation of more venturesome NS behaviour, our findings of increased suicidal ideation in the prototypic cluster, more likely are a consequence of the overall increased disease burden, that is, increased SAD symptom severity, increased HA, and comorbid depressive symptoms in this cluster. Accordingly, high HA has been associated with suicidal ideation and the total risk of suicide [71]. Furthermore, there is some evidence that suicidal ideation and self-harm attempts are observed more frequently in some personality disorders, among others, APD, which we observed to occur more frequent in the prototypic subtype [72]. However, as we only applied a one-item measure of suicidal ideation, all these speculative interpretations must be treated with caution and need further exploration.

Personality disorders, like APD, have been associated with low self-directedness (SDi) [73], another aspect that is in line with the results in this sample as the prototypic cluster, which contains more SAD individuals with APD, had lower SDi than the atypic cluster. Individuals with high SDi are described as mature, reliable, goal-oriented and self-confident while those with low SDi are frail, irresponsible and poorly integrated without external direction [15]. The former characteristics are reflected in positive outcomes e.g. wellbeing [74, 75], autonomy, self-acceptance [74] and treatment outcome in depressive patients [76] whereas the latter are connected to mood disorders [77] and higher psychopathological distress [78]. Based on the T-scores, however, both clusters are still below the healthy norm group in this dimension, indicating difficulties in goal-oriented behaviour and positive self-perception for both clusters. This is particularly true for the prototypic cluster, as it achieved a low SDi score two SDs below the norm group. Combined with highly increased HA, these characteristics might be connected to increased SAD and depressive symptom severity.

Our multivariate approach further highlights the role of age. Again, with older age might come the willingness to engage and face more novel social situations. Interestingly, SAD individuals with a German high school degree are at greater odds to be in the atypic cluster, which might be related to the lower levels of HA compared to the prototypic cluster rather than the higher NS tendencies [79]. Recent research supports this relation as SAD individuals are, for example, less likely to start or obtain a university degree or obtain postgraduate education compared to healthy and unaffected siblings [80]. Longitudinal results showcased the hampering influence of SAD symptoms on social ties and academic achievement [81]. As the prototypic cluster report higher SAD symptom severity, combined with high HA, the prototypic SAD individuals are at risk of facing difficulties in educational settings. Knowing the relationship between HA and SAD symptom severity might help to tailor appropriate school or university interventions to adequately support and address the ones in need.

On a descriptive level, it is interesting to point out, that NS oriented characteristics (e.g. having children, being in a relationship, smoking, alcohol- and substance-related disorder) and HA oriented characteristics (e.g. higher medication intake, higher comorbidity rate) reached Odds ratio’s that are cluster conform. This might be a sign for future research to highlight and assess more diverse NS and HA oriented characteristics or behaviour to fully grasp the concept and nature of the prototypic and atypic cluster of SAD.

One further important, practically highly relevant clinical implication of the proposed categorisation of SAD patients lies in a more in-depth characterization of the atypic subtype. In case of a rather superficial exploration, the characteristics of the atypic SAD patients may bear the risk of missing out a diagnosis [43]. This risk seems particularly important, as these patients might be especially open minded and approachable to therapeutic intervention. First, due to the increased approach tendency, they might be more open towards even starting a therapy. Additionally, they might more likely take an active part in confrontational therapeutic interventions, which should increase treatment efficacy (for relevance of confrontational approaches in cognitive behavioural therapy see e.g. [82]). Besides that, early intervention seems especially important to prevent the development of dysfunctional coping strategies in terms of substance misuse in atypic SAD patients.

## Conclusion

The present study provides further evidence for the heterogeneity of SAD [5, 6]. It contributes to a better understanding of SAD characteristics, and therewith can provide several important implications for the clinical routine. Our data support the existence of distinct subtypes of SAD patients. Besides a prototypic SAD subtype suffering from more severe SAD symptoms, comorbid depression, and functional impairment, there is a more NS oriented subtype with less severe symptoms, a higher level of social functioning and self-directedness. A better knowledge of the characteristic differences in prototypic behaviour, personality, coping strategies and comorbidities between the identified (and, perhaps further) subtypes can contribute to the development of effective prevention interventions [83], and promotes the conceptualization of tailored treatments.

## Supplementary Information

**Additional file 1:** **Table A.** Comparison of the compared cluster solutions.

## Data Availability

Data are available from the corresponding author on reasonable request.
