# Supplementary material for: Personality subtypes in adults with social anxiety disorder - novelty seeking makes the difference
Source: BMC Psychiatry. 2022 Dec 27;22:832. doi: 10.1186/s12888-022-04484-z (PMC9793521; doi:10.1186/s12888-022-04484-z)
Supplement: Supplementary file 1 — Additional file 1: Table A. Comparison of the compared cluster solutions. [file 12888_2022_4484_MOESM1_ESM.docx]

# Supplementary Information

| **Table A** | | | | | | |
| --- | --- | --- | --- | --- | --- | --- |
| Comparison of the compared cluster solutions. | | | | | | |
|  | | Number of extracted clusters | | | | |
|  | | 2 | 3 | 4 | 5 | 6 |
| ETA² | | .46 | .59 | .68 | .73 | .78 |
| PRE | | .46 | .24 | .22 | .15 | .17 |
| FMX | | 484.09 | 412.07 | 407.02 | 384.53 | 393.49 |
| Note: | ETA^2^ = explained variance, PRE = relative improvement of ETA^2^, FMX = best variance ratio | | | | | |
